# Pericardial Fat Is Associated with Coronary Artery Calcification in Non-Dialysis Dependent Chronic Kidney Disease Patients

**DOI:** 10.1371/journal.pone.0114358

**Published:** 2014-12-05

**Authors:** Paulo H. N. Harada, Maria E. Canziani, Leonardo M. Lima, Maria Kamimura, Carlos E. Rochitte, Marcelo M. Lemos, Lilian Cuppari, Roberto Kalil Filho, Sergio A. Draibe, Raul D. Santos

**Affiliations:** 1 Lipid Clinic Heart Institute (InCor) University of Sao Paulo Medical School, Sao Paulo, Brazil; 2 Nephrology Division, Federal University of Sao Paulo, Sao Paulo, Brazil; 3 Cardiovascular Magnetic Resonance and Computed Tomography Sector, Heart Institute (InCor) University of São Paulo Medical School, Sao Paulo, Brazil; Kaohsiung Chang Gung Memorial Hospital, Taiwan

## Abstract

Pericardial fat (PF) a component of visceral adipose tissue has been consistently related to coronary atherosclerosis in the general population. This study evaluated the association between PF and coronary artery calcification (CAC) in non-dialysis dependent chronic kidney disease (CKD) patients. This is a post-hoc cross sectional analysis of the baseline of a prospective cohort of 117 outward CKD patients without manifest coronary artery disease (age, 56.9±11.0 years, 64.1% males, 95.1% hypertensives, 25.2% diabetics, 15.5% ever smokers, CKD stage 2 to 5 with estimated glomerular filtration rate 36.8±18.1 ml/min). CAC scores, PF volume and abdominal visceral fat (AVF) areas were measured by computed tomography. The association of PF as a continuous variable with the presence of CAC was analyzed by multivariate logistic regression. CAC (calcium score >0) was present in 59.2% patients. Those presenting CAC were on average 10 years older, had a higher proportion of male gender (78.7% vs. 42.9%, p<0.001), and had higher values of waist circumference (95.9±10.7 vs. 90.2±13.2 cm, p = 0.02), PF volumes (224.8±107.6 vs. 139.1±85.0 cm^3^, p<0.01) and AVF areas (109.2±81.5 vs. 70.2±62.9 cm^2^, p = 0.01). In the multivariate analysis, adjusting for age, gender, diabetes, smoking and, left ventricular concentric hypertrophy, PF was significantly associated with the presence of CAC (OR: 1.88 95% CI: 1.03–3.43 per standard deviation). PF remained associated with CAC even with additional adjustments for estimated glomerular filtration rate or serum phosphorus (OR: 1.85 95% CI: 1.00–3.42, p = 0.05). PF is independently associated with CAC in non-dialysis dependent CKD patients.

## Introduction

Chronic kidney disease (CKD) is highly prevalent affecting roughly 7% of the general adult population and up to one in three elder individuals [1]. Despite concerns about progression of CKD from stages 1–3 to stages 4 and 5, overall mortality risk overrides the risk of end stage renal disease onset by a 20-time factor [2]. Indeed cardiovascular mortality is responsible for 50% of overall deaths and is up to 20 times more incident in CKD individuals than in the general population [3].

Coronary artery calcification (CAC), a marker of subclinical vascular disease that is highly prevalent in CKD, reliably predicts the risk for overall mortality and cardiovascular events in dialytic [4], [5] as well as in non-dialytic patients [6], [7]. The mechanisms of CAC buildup in CKD patients are multiple, and classic atherosclerotic risk factors like hypertension and dyslipidemia interact with factors related with uremia like malnutrition, inflammation [8], and disorders of mineral metabolism [9].

Pericardial fat (PF) comprises all fatty tissues in the mediastinum surrounding the myocardium, especially over the epicardial coronary track [11]. As a component of the visceral fat compartment, PF produces highly active cytokines [10]. Since there is no physical barrier between PF and coronary arteries, PF has been advocated as a paracrine promoter of CAC. This hypothesis is supported by studies in non-CKD populations associating PF respectively with CAC presence [11], [12], high-risk coronary atherosclerotic plaque features [13] and incident myocardial infarction [14]. PF has been related with coronary plaque presence even in lean individuals [15], which support the hypothesis that PF could reflect more accurately a riskier adiposity in comparison with other fatness parameters.

There is limited evidence associating epicardial and pericardial fat with CAC in CKD individuals [16], [17]. The aim of this study was to test the association of PF with CAC presence both measured by computed tomography in non-dialysis dependent CKD patients.

## Material and Methods

### Patients

This is a post hoc analysis from a prospective cohort that comprised 117 stage 2–5 non-dialysis chronic kidney disease (ND CKD) patients followed at the outpatient clinic of the Nephrology Division, Federal University of São Paulo (UNIFESP) in Sao Paulo, Brazil [6]. All patients had been previously submitted to cardiac computed tomography for CAC quantification, but just 103 had adequate images for PF reading. This study was approved by UNIFESP′s institutional review board and a written informed consent was obtained from all participants.

The exclusion criteria were: age <18 years, presence of chronic inflammatory diseases, current malignancy, HIV infection, viral hepatitis, chronic steroid usage, and previous manifestations of coronary heart disease. The majority was using angiotensin converting enzyme inhibitors (82.2%); 22.9% angiotensin receptor blockers, 77.1% diuretics, 41.5% beta-blockers, 34% patients were on statins and 4.2% on recombinant human erythropoietin. Thirty patients (29.1%) were using sevelamer, six (5.0%) calcium based phosphate binders, and six (5.0%) calcitriol.

### Clinical, Laboratory and Echocardiography Evaluation

All patients underwent clinical evaluation, where their personal and familiar previous medical history and physical exam were addressed. Diabetes was defined as a fasting glucose >126 mg/dL or current usage of any anti-diabetic medication. Hypertension was ascertained by average blood pressure over 140×90 mmHg in two subsequent measurements after 15 minutes resting in sitting position or usage of any anti-hypertensive medication. Current smoking was defined as any cigarette consumption in the last month and ever smoking as any previous daily cigarette sustained consumption for at least 6 months. Body weight (kg), height (m), waist circumference (cm), as well as systolic and diastolic arterial pressure (mmHg) were measured as previously described [6]. The body mass index (kg/m^2^) was calculated. Blood samples were drawn in the fasting state. The biochemical parameters including serum creatinine, ionic calcium, phosphorus, alkaline phosphatase, lipid profile, hemoglobin, albumin and intact parathyroid hormone (iPTH) were measured by chemiluminescence immunoassay (Immulite; DPC-Biermann, Bad Nauheim, Germany). High-sensitivity C-reactive protein (CRP) was measured by an immunometric assay (Immunolite, Immunometric Assay, CA, USA). Proteinuria was directly measured by 24-hour urine samples and the glomerular filtration rate (eGFR) was estimated by the simplified “Modification of Diet in Renal Disease” (MDRD) equation. Abnormal proteinuria was defined as excretion >300 mg/24 hours. The diagnosis and classification of CKD was determined according to KDIGO guidelines criteria [18].

A two-dimensional color Doppler echocardiogram was performed in each patient according to the recommendations of the American Society of Echocardiography [19] using the Philips HDI 5000 equipment (Royal Philips Electronics, Netherlands). Left ventricular concentric hypertrophy was defined as the presence of left ventricular mass hypertrophy and abnormal concentric remodeling. Relative wall thickness >0.42 was considered abnormal and a left ventricular mass index >115 g/m^2^ and >95 g/m^2^ respectively in men and women was considered elevated [20]. Left ventricular dysfunction was defined by an ejection fraction ≤55%.

### Coronary Artery Calcification Evaluation

CAC was quantified by multi-slice computed tomography (LightSpeed Pro 16; GE Healthcare, Milwaukee, WI). The tomographic axial slices were acquired with a 2.5 mm thickness, prospective acquisition and synchronization to EKG, with a 0.4 seconds rotation, temporal reconstruction by 6 frames per second, and W-ray tube voltage of 120 kV and 350 mA. CAC was quantified as previously described by Agatston et al. [21]. Any CAC>0 was categorized as CAC presence. CAC severity was stratified in: 0;>0–100 and >100 Agatston Units (AU).

### Pericardial Fat, Visceral and Subcutaneous Abdominal Fat Quantifications

**Figure 1:**
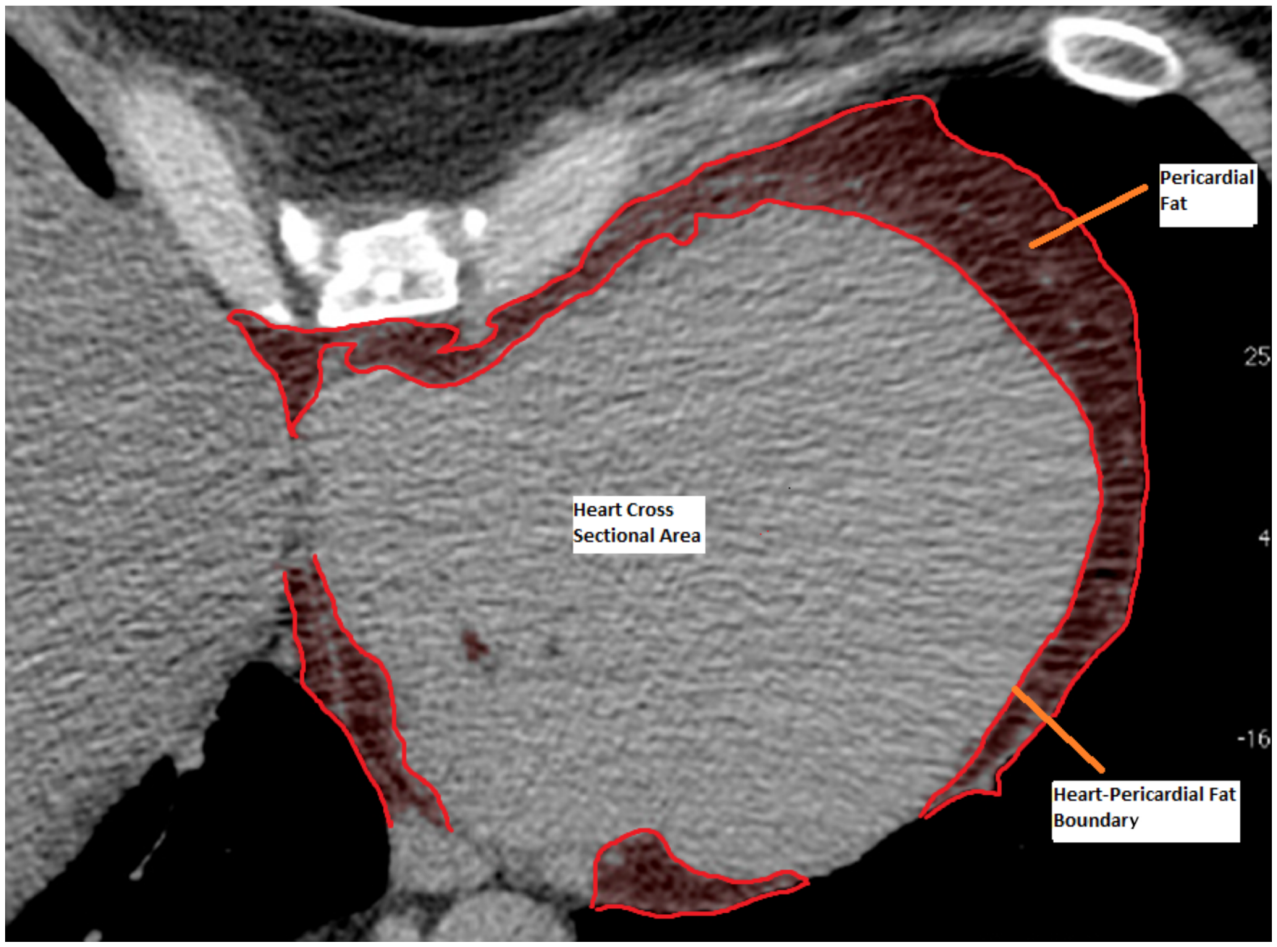
Cross sectional (axial) area of pericardial fat (PF). Colored image outlining axial PF area boundaries. Cardiac silhouette in grey, within PF and labeled.

**Figure 2:**
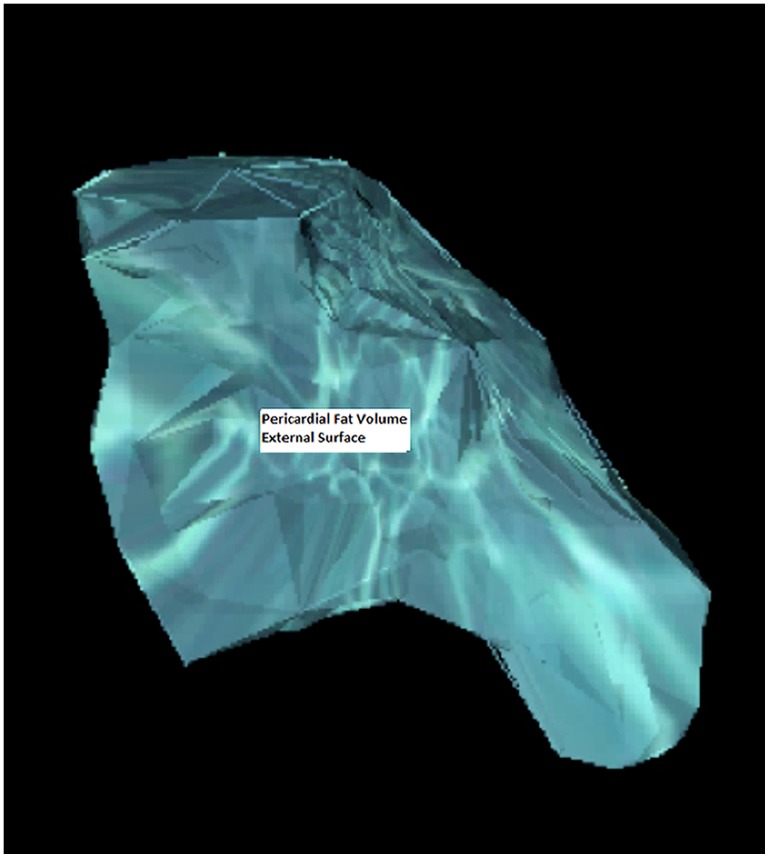
Pericardial fat volume, antero-posterior external view of its tridimensional reconstruction.

**Figure 3:**
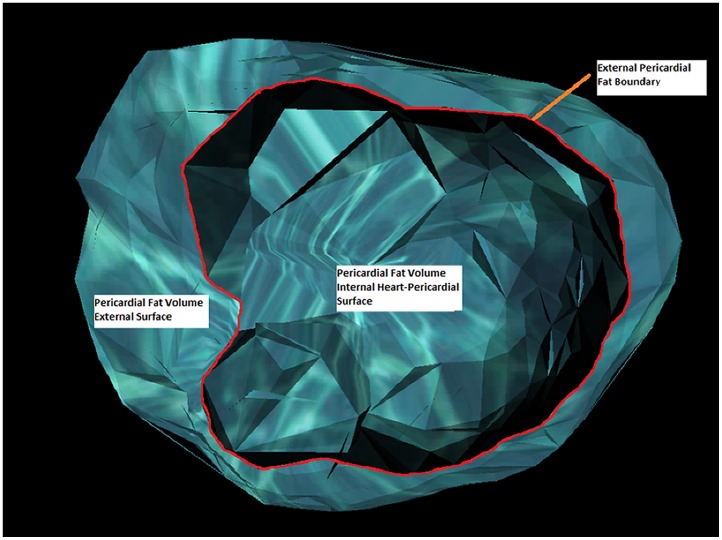
Pericardial fat (PF) volume, cranio-caudal view of internal and external surface of its tridimensional reconstruction. Exclusion of the most cranial axial surface to display the internal cavity of PF volume.

The PF was analyzed at the Lipid Clinic of the Heart Institute (InCor) University of Sao Paulo Medical School Hospital (HCFMUSP) with supervision from the Cardiac Tomography and Resonance Service at the same hospital. The images obtained for CAC measurement were recovered and uploaded on an I- Mac Apple computer with and Intel Core i3 processor running MacOSx version 10.6. The Osirix Imaging Software (Pixmeo, Switzerland), open source version 3.9 [22] was used for fat quantification by a semi-automatic process developed at InCor. The PF volume (in cm^3^) was calculated from measurements of each PF axial slice area (in cm^2^). In each tomography slice a semi-automatic selection by radiodensity window ranging from −30 to −200 Hounsfield units using the limiar technique (threshold) was made. For the segmentation, a tool from the Osirix software, which detects tissues within the mentioned thresholds surrounding the selection point was used. By this technique fat areas demarked in each axial slice, and fat outside PF borders were manually delimitated to display the final axial area (Figure 1). The PF boundaries were defined as: 1-superior, pericardial reflexion near the pulmonary artery and bellow aortic arch; 2-inferior, diaphragmatic transition; 3-posterior the line between the right and left main bronchi; and 4-anterior, the internal limit of anterior thoracic wall. The PF volume was then obtained by the sum of each axial slice area weighted by its own thickness, from the diaphragm up to the last slice registering mediastinal fat, and calculated automatically by the command “compute volume” (Figures 2 and 3). In each patient, PF was measured by two experienced observers. For accuracy evaluation, intra-observer and inter-observer intra-class correlations were done for each image. The inter-observer intra-class correlation was 0.99 (p<0.01). Indexation of PF by body surface area using the DuBois method was not done due to the high correlation of the former with absolute PF values (r = 0.98 p<0.01).

**Figure 4:**
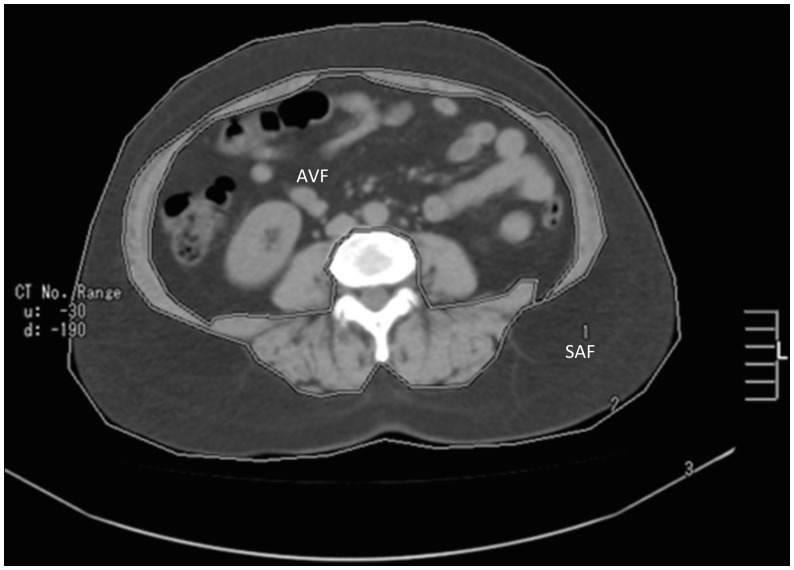
Cross sectional (axial) area of subcutaneous abdominal fat (SAF) and abdominal visceral fat (AVF) on L4–L5 vertebrae topography. Each area comprehends contiguous images within outlined boundaries.

Abdominal visceral fat (AVF) and subcutaneous abdominal fat (SAF) areas (cm^2^) were measured by computed tomography (Helical Picker PQ 5000, Cleveland, OH, USA) at the level of the 4^th^ and 5^th^ (L4 and L5; Figure 4) lumbar vertebrae. All subjects were examined in the supine position with both arms stretched above the head, and 10 mm slices were measured at the L4–L5 levels. Visceral and subcutaneous fat areas (in cm^2^) were obtained by delineating and computing the adipose tissue surface using an attenuation range of −150 to −50 Hounsfield units.

### Statistical Analysis

Data normality was tested by the Kolmogorov-Smirnov test. Normally distributed data were expressed as mean ± standard deviation, while non-normal data were expressed as median and interquartile ranges. Categorical variables were depicted as percent frequency (proportions). Chi-square test or Fisher's exact test were used to compare categorical variables when pertinent. Continuous variables were analyzed by Student's t test or by Mann-Whitney's test. The correlation between PF with the other markers of adiposity: waist circumference, BMI, AVF and SAF was tested by Pearson's test. Univariate and multivariate logistic regression analysis were performed to evaluate the association between presence of CAC and other variables. PF was expressed in standard deviation (SD) units rather than cm^3^ for better understanding the variability derived by PF range values. Associations were described through the odds ratios (OR) and 95% confidence intervals (CI). The direct comparison of CAC presence discrimination by PF and AVF was made by their individual receiver operating characteristic curves. P values <0.05 were considered statistically significant. The analyses were made using, the SPSS software for Macintosh (IBM SPSS Statistics, Version 20.0. Armonk, NY: IBM Corp).

## Results

Table 1 shows the demographic and clinical characteristics of the studied population. Patients were predominantly middle-aged men, 25% had diabetes, the majority had hypertension. Obesity and overweight were found respectively in 27.2% and 59.2% of subjects. According to the CKD classification, 15 patients (14.6%) were in stage 2, 40 (38.8%) in stage 3, 43 (41.7%) in stage 4, and 5 (4.9%) in stage 5. Proteinuria was found in 44 (42.7%) of the patients.

**Table 1:** Clinical, laboratory and echocardiographic characteristics in the whole study population and according to the presence or not of coronary artery calcification (CAC).

Variables	All	CAC = 0	CAC>0	P value
N (%)	103 (100)	42 (40.8)	61 (59.2)	
Age (years)	56.9±11.0	50.8±11.2	61.1±8.6	<0.001
Gender (male)	64.1%	42.9%	78.7%	<0.001
Diabetes	25.2%	16.7%	31.1%	0.10
Hypertension	95.1%	92.9%	96.7%	0.40
Current Smoker	15.5%	19%	13.1%	0.41
Ever Smoker	51.5%	40.5%	59%	0.07
Body Mass Index (kg/m^2^)	26.7±4.9	26.1±5.6	27.1±4.3	0.29
Waist Circumference (cm)	93.6±12.1	90.2±13.2	95.9±10.7	0.02
SAP (mmHg)	128±16	126.8±16.2	128.7±16.6	0.55
LVEF (>0.55)	92.2%	97.6%	88.5%	0.14
LVCH	18.4%	11.9%	23%	0.16
Total cholesterol (mg/dl)	184±37	178±32	188±40	0.19
HDL cholesterol (mg/dl)	51±14	52±13	51±15	0.61
LDL cholesterol (mg/dl)	101±28	97±26	104±29	0.18
Triglycerides (mg/dl)	160 (97–208)	121. (99–182)	147 (95–215)	0.63
Creatinine (mg/dL)	2.19±0.84	2.18±0.83	2.20±0.85	0.89
eGFR (ml/min)	36.8±18.1	37.9±18.2	36.0±18.2	0.40
Proteinuria (mg/L)	0.24 (0.00–0.76)	0.2 (0.00–0.76)	0.24 (0.00–0.77)	0.69
Hemoglobin (g/L)	12.8±1.8	12.8±1.70	12.8±1.9	0.98
CRP (mg/dL)	0.27 (0.11–0.70)	0.22 (0.08–0.66)	0.28 (0.15–0.82)	0.15
Phosphorus (mg/dL)	3.8±0.7	3.67±0.73	3.81±0.72	0.32
Ionic Calcium (mmol/L)	1.3±0.1	1.28±0.06	1.29±0.06	0.68
Alkaline Phosphatase (U/L)	80 (66–101)	80 (59.5–104)	81 (68–100)	0.31
iPTH (pg/ml)	103 (62–189)	106 (67.5–197.8)	103 (56.5–160.5)	0.61
PF (cm^3^)	189.9±107.2	139.1±85.0	224.8±107.6	<0.01
AVF (cm^2^)	93.3±81.4	70.2±62.9	109.2±81.5	0.01
SAF (cm^2^)	179.2±94.5	180.5±108.1	178.4±84.8	0.91

Values displayed in mean ± standard deviation or median (percentile 25–percentile 75). SAP-systolic arterial pressure; LVEF-left ventricular ejection fraction; LVCH-left ventricular concentric hypertrophy; eGFR-estimated glomerular filtration rate; CRP-high sensitivity C reactive protein; iPTH-intact parathormone; PF-pericardial fat; AVF- abdominal visceral fat; SAF-subcutaneous abdominal fat.

The PF was related with age (r = 0.43, p<0.001) and was higher in men (220.4±110.5 vs.135.4±76.0 cm^3^ in women, p<0.001). Regarding other adiposity markers, PF volume correlated with AVF area (r = 0.82, p<0.01), waist circumference (r = 0.67, p<0.01), BMI (r = 0.57, p<0.01) and SAF area (r = 0.31, p<0.01). PF was not related with eGFR (r = 0.05, p = 0.65) and was not different in diabetic compared to non-diabetic patients (respectively, 200.4±110.5 and 186.4±106.5 cm^3^, p = 0.58).

Overall, 61 patients (59.2%) had CAC and among those the median (interquartile ranges- IQR) calcium score was 219 (IQR 25.5–756.0) AU. Forty-two patients (40.8%) had no coronary calcification (CAC score  = 0), in 23 patients (22.4%) CAC score was between 1–100 AU, and in 38 (36.9%) CAC score was >100 AU. The distribution of PF within CAC = 0, CAC>0–100 and CAC>100 groups were respectively, 139.1±85.0; 221.0±119.3 and 227.1±101.5 cm^3^ (p<0.01).

Table 1 shows the comparison between patients presenting or not CAC. Those with CAC were older, had a greater prevalence of male gender, and had higher values of waist circumference (p<0.05). There were no differences between the groups regarding previous history of hypertension, smoking, diabetes, BMI, systolic blood pressure values, eGFR as well as laboratory and echocardiographic parameters. Patients with CAC had higher values of PF volume (p<0.01) and AVF area (p = 0.01). However, there was no difference in SAF areas between the two groups.

**Figure 5:**
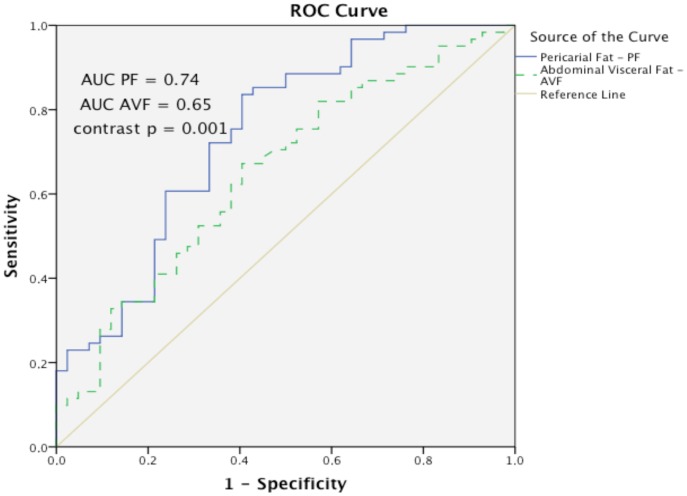
Receiving operating characteristic curves (ROC) for pericaridal fat (PF) volumes and abdominal visceral fat (AVF) for coronary artery calcification (CAC) presence.

Table 2 shows the univariate associations of CAC presence. CAC was positively related to age, male gender, smoking, waist circumference, PF volumes and AVF areas. Table 3 shows that PF was independently associated with CAC presence (OR: 1.88 CI: 1.03–3.43) after adjusting for age, gender, diabetes, smoking and, left ventricular concentric hypertrophy. PF remained associated with CAC even with additional adjustments for estimated glomerular filtration rate or serum phosphorus. Figure 5 demonstrates the individual better discriminatory effect of PF volume versus AVF area for CAC presence (p = 0.001).

**Table 2:** Univariate associations with the presence of coronary artery calcification (CAC) by logistic regression analysis.

Variables	OR	95% CI	p value
Age (years)	1.11	(1.06–1.17)	<0.01
Gender (male)	4.92	(2.07–11.70)	<0.01
Ever Smoking	2.12	(0.95–4.71)	0.07
Pericardial Fat (per SD)	2.85	(1.63–4.98)	<0.01
Abdominal Visceral Fat (per SD)	1.80	(1.13–2.89)	0.01
Waist Circumference (cm)	1.63	(1.06–2.51)	0.03

SD- standard deviation.

**Table 3:** Association of pericardial fat with coronary artery calcification presence after multiple adjustments.

	OR	95% CI	p value
**Model 1**			
Pericardial Fat (SD)	1.88	1.03–3.43	0.04
**Model 2**			
Pericardial Fat (SD)	1.89	1.05–3.42	0.03
**Model 3**			
Pericardial Fat (SD)	1.85	1.00–3.42	0.05

Model 1: adjusted for age, gender, smoking, diabetes and left ventricular concentric hypertrophy; Model 2: Model 1+ eGFR; Model 3: Model 1+ serum phosphorus; OR: Odds Ratios; CI: confidence intervals.

## Discussion

This study addressed the association of PF volume with CAC presence measured by computed tomography in non-dialysis dependent CKD patients. PF was independently associated with CAC even after adjustment for parameters previously related with CAC in this population. The results suggest a role of PF, a component of the visceral fat compartment adjacent to the heart, on the pathophysiology of coronary vascular calcification development in CKD patients.

### Independent Association of PF with CAC

PF is considered as part of the visceral adipose tissue compartment [10], and its association with CAC could have signaled only a surrogate of the deleterious effects caused by the more profuse visceral abdominal fat component upon the coronary arteries. Indeed in this study there was a strong correlation between PF volume and adipose visceral mass both measured by computed tomography. Increased abdominal visceral adipose tissue mass has been associated with subclinical coronary atherosclerosis in subjects with normal kidney function [11], [23] and with CAC in CKD patients [24]. Abdominal visceral adipocytes secrete adipocytokines that induce vessel inflammation while at the same time lack adequate adiponectin secretion, a hormone previous shown to inhibit osteoblast formation [25]. Indeed these characteristics were also shown for pericardial adipocytes [26], [27].

Notwithstanding the known endocrine effects of abdominal visceral adipocytes on the coronary arteries [23], in the present study, PF discriminated CAC presence better than AVF as was observed by a greater area under the receiver operating curve. This suggests that PF could contribute to CAC development by both endocrine and paracrine effects [28]. A paracrine effect would mean a direct contiguous effect where the release of cytokines from pericardial fat could reach the coronary wall by diffusion, go directly into *vasa vasorum* or through systemic circulation [29]. As PF volumes and AVF areas presented an elevated collinearity it was not adequate to use them concomitantly in any statistical model.

### Previous Evidence of the PF and CAC Association in CKD Patients

In CKD patients Turkmen et al. found a positive association of epicardial fat, a subset of PF located below the visceral pericardial sac, with CAC presence in peritoneal dialysis patients [16]. In that study epicardial fat was associated with CAC independently of BMI. D′Marco et al. found in a post hoc subgroup analysis of the Renagel in New Dialysis (RIND) study a positive association between epicardial fat and overall mortality in incident hemodialysis patients [30]. In non-end stage CKD patients only one study had addressed the relation of CAC, laboratory biomarkers, diabetes, and renal function with epicardial fat measured by computed tomography [17]. Kerr et al. evaluated 94 pre-dialysis stage 3–5 CKD patients, mean age: 63.7 years, 56% male, and mean eGFR of 25.1±11.9 mL/min/1.73 m^2^. In that study CAC and serum levels of interleukin-6 were associated with epicardial fat volume (the dependent variable). The current study addresses this association by two different aspects: first, PF that comprises not only epicardial fat, that is located directly over the coronary arteries, but also within the rest of the pericardial sac was measured; second, we depart from PF to CAC (the dependent variable) as the physiopathological premise is PF being a promoter of CAC rather than the opposite.

### Clinical Implications and Further Research

The results of this study reinforce the possible role of ectopic fat accumulation on the rather complex pathophysiology of CAC in CKD patients [8], [9]. It is likely that PF contributes to CAC independently of abdominal fat and its determination by computed tomography might have clinical implications since CAC is an independent marker of cardiovascular morbidity and mortality in the CKD population [4]–[7]. However, larger and prospective studies are necessary to prove this point. Also, it would be necessary to show that if reduction of PF could alter the course of cardiovascular disease in this population.

### Study Limitations

This is a post-hoc analysis of a cohort of CKD patients. However, all the data including clinical, laboratory and computed tomography parameters were collected concomitantly. So far there is no gold standard in the literature to adequately quantify the different components of thoracic ectopic adipose tissue that comprises pericardial, epicardial and intra-thoracic components. Therefore, the comparison of this study with previous results should be made with care. However, it is important to reinforce the high reliability of our measurement technique, confirmed by the high inter-observer index.

On the multivariate analysis the confounding effect of hypertension for CAC is paramount. As more than 95% of the sample was hypertensive, using simply categorical labeling would not identify all the differential spectrum of exposition burden. Systolic arterial pressure as well would not discriminate this hemodynamic burden, since the sample had a good average control due to antihypertensive medication. So, concentric hypertrophy was used as marker of hypertension burden in our population. Indeed Ertük [31] et al. found a good association of echocardiographic ventricular hypertrophy with systolic burden by ambulatory arterial blood pressure monitoring in a CKD dialysis sample. Also Ehara et al. [32] demonstrated that the absence of concentric hypertrophy was associated with CAC absence in a non-CKD population referred for suspected coronary artery disease. These data support the usage of concentric hypertrophy as a confounder for the studied association. Finally, the nature of this study only allows showing association and not causality.

## Conclusion

PF volume measured by computed tomography was associated with CAC presence in non-dialysis dependent CKD population. Further studies are required to evaluate this association.
